# A Manual of the Practice of Medicine

**Published:** 1891-02

**Authors:** 


A MANUAL OF THE PRACTICE OF MEDICINE. By Frederick Taylor, M. D., F. R. C. P., Physician to, and Lecturer on Medicine at Guy's Hospital, Physician to the Evilina Hospital for Sick Children and Examiner in Materia Medica, etc., at the University of London. P. Blakiston, Son & Co., Publisher, Philadelphia, 1890.

This is a Hand-Book of the practice of medicine, better suited to the student and country practitioner than to those able to study more elaborate works. Of course, no author can do justice to science or to himself when he undertakes to explain his subject in too concise a manner. It is impossible to give a "complete account of the present state of medical practice" in a volume of 859 pages.

There is this, however, to commend the work: good space is given to the presentation of symptoms—a thorough knowledge of which is invaluable in diagnosis. Every symptom, insignificant as it may seem, has a certain value in making out the case. No classification of diseases can be perfect, until the science of medicine itself is perfect. We ought not therefore to be in too great haste to condemn the author who arranges his subjects according to his opinion of pathology. We do not think that Etiology received sufficient attention by Dr. Taylor, but upon the whole, it is a very good book and there is a place for it in the Medical Library.

